# Can Developments in Tissue Optical Clearing Aid Super-Resolution Microscopy Imaging?

**DOI:** 10.3390/ijms22136730

**Published:** 2021-06-23

**Authors:** Paweł Matryba, Kacper Łukasiewicz, Monika Pawłowska, Jacek Tomczuk, Jakub Gołąb

**Affiliations:** 1 Department of Immunology, Medical University of Warsaw, 02-097 Warsaw, Poland; jacektomczuk1997@gmail.com (J.T.); jakub.golab@wum.edu.pl (J.G.); 2 The Doctoral School of the Medical University of Warsaw, Medical University of Warsaw, 02-097 Warsaw, Poland; 3 Laboratory of Neurobiology, BRAINCITY, Nencki Institute of Experimental Biology of Polish Academy of Sciences, 02-093 Warsaw, Poland; m.pawlowska@nencki.edu.pl; 4 Department of Molecular, Cell and Developmental Biology, University of California Santa Cruz, Santa Cruz, CA 95064, USA; kacper.lukasiewicz@gmail.com; 5 Institute of Experimental Physics, Faculty of Physics, University of Warsaw, 02-093 Warsaw, Poland

**Keywords:** tissue clearing, clearing agents, optical clearing, light sheet, super-resolution, CUBIC, DISCO, CLARITY

## Abstract

The rapid development of super-resolution microscopy (SRM) techniques opens new avenues to examine cell and tissue details at a nanometer scale. Due to compatibility with specific labelling approaches, in vivo imaging and the relative ease of sample preparation, SRM appears to be a valuable alternative to laborious electron microscopy techniques. SRM, however, is not free from drawbacks, with the rapid quenching of the fluorescence signal, sensitivity to spherical aberrations and light scattering that typically limits imaging depth up to few micrometers being the most pronounced ones. Recently presented and robustly optimized sets of tissue optical clearing (TOC) techniques turn biological specimens transparent, which greatly increases the tissue thickness that is available for imaging without loss of resolution. Hence, SRM and TOC are naturally synergistic techniques, and a proper combination of these might promptly reveal the three-dimensional structure of entire organs with nanometer resolution. As such, an effort to introduce large-scale volumetric SRM has already started; in this review, we discuss TOC approaches that might be favorable during the preparation of SRM samples. Thus, special emphasis is put on TOC methods that enhance the preservation of fluorescence intensity, offer the homogenous distribution of molecular probes, and vastly decrease spherical aberrations. Finally, we review examples of studies in which both SRM and TOC were successfully applied to study biological systems.

## 1. Introduction

Significant developments in microscopy instrumentation have recently pushed the field of biomedical imaging beyond numerous limits. Depending on the nature of the scientific question being asked, it is currently possible to perform the imaging of biological specimens with a staggering resolution greater than 1 Å [1,2] (e.g., using a transmission electron microscope) or by combining recent, advanced protocols of tissue optical clearing (TOC) [3] and selective plane illumination microscopy (SPIM, also known as light-sheet fluorescence microscopy, LSFM) [4], with single-cell resolution (10–100 μm) within entire murine bodies [5]. Although the value of electron microscopy studies cannot be overestimated, undoubtedly this set of techniques is not free from several major disadvantages from the perspective of biologists, with the technically challenging process of sample preparation and limited choice for their labeling being perceived as the major weaknesses. As “nature abhors a vacuum”, this obvious limitation of the electron microscopy stimulated developments of light microscopy in a direction that enables successful imaging of nanometer-sized structures, while retaining the vast majority of its natural advantages, e.g., cell-specific staining approaches, ease of sample preparation and the possibility to perform live imaging, just to name a few.

As a result, the last two decades have brought tremendous progress in super-resolution microscopy (SRM) [6,7], a field that now makes it feasible for biologists to break Abbe’s diffraction barrier (~250 nm in the focal plane and ~550 nm in the z direction) during the imaging of both live and fixed specimens [8]. In brief, SRM refers to a set of techniques allowing microscopical structures beyond the resolution of a conventional optical microscope, limited by the diffraction of visible light, to be resolved. To improve resolution, SRM techniques adapt a variety of approaches, such as: the increase of effective numerical aperture (e.g., 4Pi microscope), nonlinear response to excitation of fluorophores (e.g., STED, RESOLFT), total internal reflection (e.g., TIRF), single molecule localization (e.g., PALM, STORM) and nonuniform illumination patterns (e.g., SIM). Furthermore, there are multiple computational techniques which increase the resolution during post-processing, such as: deconvolution, pixel reassignment in image scanning microscopy or more sophisticated image reconstruction algorithms, including those employing machine learning. Another approach to resolve microscopical structures in the specimen is by bypassing the diffraction limit with expansion microscopy (ExM), which might be listed as either a TOC (due to resultant transparency) or SRM technique.

The robustness and importance of SRM developments (additionally stimulated by a 2014 Nobel Prize in Chemistry awarded to Betzig, Hell and Moerner) for the biomedical community is represented by the number of original SRM techniques (vide infra) that have already been followed by a plethora of their modifications and applications to decipher specific biological problems [9]. Nonetheless, even such a powerful approach does not currently come without flaws, including: (1) incompatibility with imaging of, nanoscopically speaking, thick (>50 μm) tissue slices, (2) rapid photobleaching of fluorophores and (3) insufficiently strong fluorescence signals obtained after the expansion of microscopy-based approaches [8,10].

It seems that the ultimate application of SRM would have been imaging of entire, millimeter-thick organs within a reasonable timeframe, with sustainable fluorescence intensity and comparable resolution across the entire imaging depth. All of these might be achieved when combined with SPIM and TOC to guarantee a high imaging speed with low photobleaching and sample transparency (deep tissue imaging), respectively. Noteworthy, the first of such studies have already started to emerge. By combining lattice light sheet and expansion microscopy (that transparentize tissue by turning it into an isotropically enlarged sample with literally 99% water content), Gao et al. [11] imaged and presented the most detailed map of dopaminergic neurons-associated presynaptic sites within the entire brain of an adult *Drosophila melanogaster* with SRM precision maintained across the whole sample. Similarly, by utilizing classical, nonexpansion-based TOC combined with spinning disk confocal microscopy, Lin et al. [12] recently reported a 20-nm lateral resolution in 200-μm-deep samples of *D. melanogaster* brain. Inevitably, with such significant progress witnessed in the field of biomedical imaging in recent years, the addition of TOC to SRM-based experiments will shortly become the standard that further expands the utility of this imaging approach.

In this review, we aim to present how the tremendous progress in the development of TOC methods might support SRM-based studies and which TOC approaches should be perceived favorably in overcoming recognized SRM shortages. First, we briefly discuss the general characteristics of both TOC and SRM and provide references to recent review articles that cover and update these topics separately. Next, we present how the application of a proper TOC method can either (1) aid studies that utilize SRM or (2) make SRM imaging of the millimeter-thick samples feasible, and finally, we present results on how these two novel approaches were already combined.

## 2. Overview of the Existing Methods

### 2.1. Tissue Optical Clearing (TOC) Techniques

**Figure 1:**
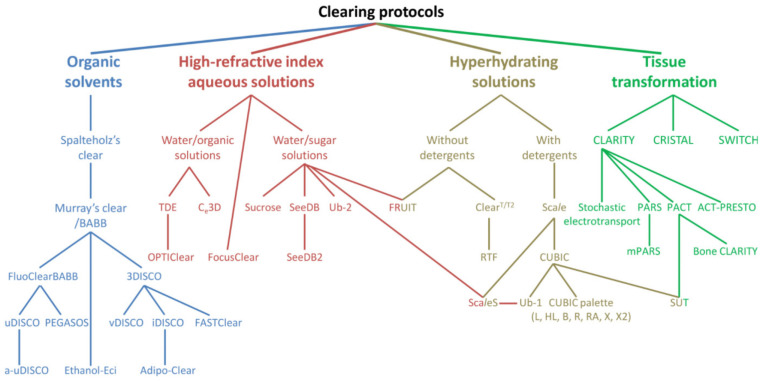
Arborization of the family of TOC protocols. The diagram represents four broad, chemical categories of TOC along with major TOC techniques. Reproduced from Matryba et al. [19] under the terms of the Creative Commons CC-BY-NC license.

Over the past decade, interest in the development and application of TOC has increased tremendously [13,14], resulting in the publication of dozens of original TOC methods along with hundreds of their optimizations (Figure 1). While initially most work focused on whole-brain imaging [15,16,17,18], by now, TOC has been applied to every organ of laboratory rodents [19,20] with multiple studies presenting completely new biomedical imaging opportunities to study, e.g., implant–tissue interface [21] or even amorphous samples, sputum from patients suffering from cystic fibrosis [22] or blood clots [23], in particular.

**Figure 2:**
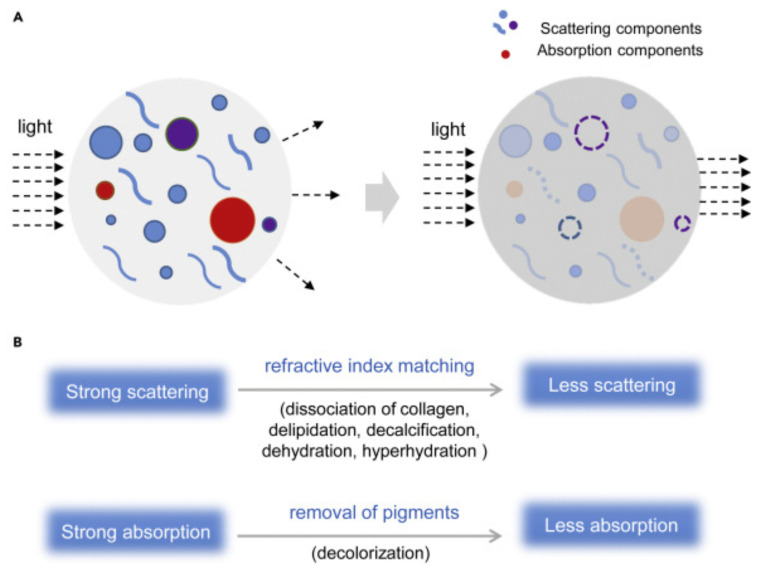
(**A**) Scheme presenting basic physicochemical mechanisms of TOC. (**B**) TOC relies mainly on the reduction of light scattering (achievable though delipidation, dehydration or hy-perhydration, decalcification and dissociation of collagen fibers) and absorption. Reproduced from Yu et al. [25] under the terms of CC BY-NC-ND 4.0 license.

Irrespective of the TOC method used, this set of techniques aims at turning opaque samples into translucent, light-permitting ones (Figure 2) [24,25,26]. The resulting transparency enables imaging deep into the tissue which is further advanced when combined with SPIM technology to look at the larger focal areas with reduced photobleaching (“plane-by-plane” imaging), then with confocal microscopy (“point-by-point” imaging). Although when categorized based on the chemical nature of the main chemical used, almost all TOC methods fall into four general categories: organic solvents, high-refractive index aqueous solutions, hyperhydration solutions and tissue transformation techniques [19,27]. Newer, advanced TOC protocols often apply chemicals from distinct TOC approaches [28,29,30] and as such take advantage of specific strengths from each of their forebears. For example, the PEGASOS method [28], even suitable for whole-body clearing (which proves its wide applicability and compatibility with different organs of interest), consists of (1) a decalcification step with EDTA, (2) Quadrol-based tissue decolorization, (3) tert-butanol-mediated tissue delipidation and, finally, (4) refractive index (RI) matching with organic solvents. Thus, the original chemical categories of TOC, although important to help understand the basic principles behind TOC, begin to deteriorate. In an application-based manner, crucial for proper combination with the specific SRM approach, chemical and physical mechanisms of TOC play a decisive role. These include decolorization, delipidation, dehydration or hyperhydration, decalcification and dissociation of collagen fibers and have been recently broadly discussed by the Zhu group [25] and us [19]. Briefly, decolorization (of heme, melanin, chlorophyll and lipofuscin) and delipidation enhance light penetration through the sample, while delipidation additionally acts as a permeabilization buffer that aids in the penetration of probes. Naturally, a significant RI mismatch occurs within tissues both intracellularly and extracellularly due to the large difference between the RI of water (~1.33, that constitutes 70–80% of tissue weight) and proteins (>1.45). Thus, a decision usually must be made whether it is advantageous either to hyperhydrate the sample (i.e., reduce the overall RI closer towards that of water and dilute the remaining light-absorbing molecules) or dehydrate. Organoleptically determined [31], proven by light-transmission experiments, dehydration will usually result in a higher transparency when compared to hyperhydrating solutions [20,32,33]. This, however, suffers from significant tissue shrinkage [34] that might prevent successful SRM imaging. A combination of tissue transforming approaches that rely on the generation of a crosslinked mesh of swellable polyelectrolyte hydrogels, followed by extensive hyperhydration (that dilutes all of the scatterers present in a tissue and homogenizes its RI to match that of a water), is core to the expansion microscopy-based TOC [35]. Expansion microscopy is a very promising method to be utilized during the SRM experiments as it not only allows for volumetric imaging due to the obtained transparency of the sample but also increases imaging resolution during physical enlargement of tissue components (as the expansion microscopy is almost always discussed in the literature as an unique branch of tissue treatment, not directly included in the TOC family, we decided not to focus on this set of techniques later in this review). Finally, a process of decalcification (predominantly performed with EDTA [28,36]) is important both from the whole-body imaging perspective (to remove otherwise insufficiently transparent skeleton) and for researchers that apply SRM to tissues affected by necrosis (it should be noted that generation of calcium deposits is a general, physical process that occurs in every necrotic tissue area).

### 2.2. SRM Techniques

SRM refers to a set of techniques sharing the same goal: to achieve optical imaging with a spatial resolution beyond the diffraction limit that is about half the wavelength of light or a few hundred nanometers (Table 1). This limit was discovered by Abbe in 1873 and for a long time was thought to be unsurpassable. However, Abbe’s reasoning is based on several assumptions including uniform illumination as well as the linear and stationary response of the fluorophores. By breaking one or more of them, a higher imaging resolution can be achieved. For instance, stimulated emission depletion (STED) microscopy exploits nonuniform illumination in order to bleach a ring-shaped area around the point that is to be imaged.

Recent years brought us many new SRM techniques and examples of their application (reviewed, among others, by Schermelleh et al. [37], Vangindertael et al. [8], Sigal et al. [38] and Huang et al. [39]). In particular, an excellent comparison of the currently available SRM techniques and their extended specification is provided in the review by Schermelleh et al. [37]. Due to space limitations, we will focus here on SRM techniques with a potential to be used together with TOC techniques. Examples of the application of TOC techniques for these SRM approaches are mentioned in detail in the following sections.

In general, the axial resolution of an optical microscope is several times worse than the lateral one. For this reason, many methods for high-resolution imaging focus on improving the Z-sectioning. This includes total internal reflection fluorescence microscopy (TIRF) that utilizes an evanescent wave to illuminate only around the 100-nm thin section of the specimen on the glass–water interface. However, TIRF microscopy is inherently limited to thin samples. One of the early SRM techniques relies on increasing the effective numerical aperture by using two opposing lenses, namely the 4Pi microscope [40,41]. Although spherical aberrations resulting from a refractive index mismatch were mentioned as a challenge for the 4Pi microscopy [40], to our knowledge, this SRM approach was never used together with TOC techniques.

Nonuniform illumination can also be used to enhance lateral resolution. In the family of methods known as structured illumination microscopy (SIM), the image is collected by illuminating the object with several different patterns generated from a coherent light source, i.e., a laser beam (a simple example is a set of fringes under several different angles) [8]. Mathematically, each pattern gives access to a different set of spatial frequencies, and a combination of several patterns can cover a larger range than uniform illumination, corresponding to a higher resolution. SIM is fast (only a few frames are needed for acquiring one super-resolved frame) and does not require special sample preparation. Since light interference is sensitive to phase, the refractive index homogeneity along the optical path is crucial to maintain a highly modulated illumination pattern necessary for high-quality images [41]. Indeed, it was shown TOC can improve image quality and resolution in SIM [42,43,44].

To enable the imaging of large samples, the idea of exploiting nonuniform illumination has been combined with LSFM. In fact, conventional LSFM axial resolution already depends on the thickness of the light sheet and can be higher than the diffraction limit of the imaging objective. There have been attempts to improve this further by shaping the illumination beam: for instance, by using Bessel beams. The best known is probably the lattice light-sheet microscopy developed by Betzig in 2014 [45]. While the original lattice light sheet was developed for live cell imaging, it was later combined with ExM to achieve an effective resolution of less than 100 nm [11].

STED and related methods (i.e., RESOLFT [54]) employ nonuniform illumination in scanning mode. In STED, the specimen is illuminated with two laser beams: the excitation beam stimulates spontaneous fluorescence emission in the focus region, while the donut-shaped depletion beam depletes fluorescence in the surrounding region through stimulated emission. As a result, fluorescence is emitted only in the local intensity minimum of the depletion laser, which generates an image with lateral resolution as high as 50 nm [37]. This kind of approach to super-resolution is known as ‘point spread function engineering’ (PSFE). While the penetration depth of STED is greater than that of most other SRM methods, it is still limited to several tens of microns, and tissue clearing has been proposed as a way to increase it. There are several interesting applications of TOC for STED microscopy that will be described later on [55].

While the methods described above rely heavily on modifying the illumination beam, another approach is to modify the fluorescence of the specimen. In single molecule localization microscopy (SMLM, [8]), repeated bleaching or switching off the emissive fluorophores is employed to keep only a very small subset of the fluorophore population activated at the same time, which allows for finding the precise location of each particle of fluorophore. Originally, two methods were proposed, differing in the mechanism of switching the fluorescence on and off: photo-activated localization microscopy (PALM), which utilizes photoactivation with UV light, and stochastic optical reconstruction microscopy (STORM), which originally used the photo switching of activator and reporter dye-pairs, and later, self-switching dyes in a suitable chemical environment [56]. Zwettler et al. showed that combining expansion microscopy, which increases tissue transparency, with SMLM (Ex-SMLM) significantly improves the resolution in comparison to SMLM only [57]. While SMLM offers superior resolution, it is also demanding, as precise localization of a sufficient number of molecules requires acquiring thousands of frames. This requirement is relaxed in super-resolution optical fluctuation imaging (SOFI), where correlations are computed between adjacent pixels [49]. This increases the image resolution without localizing single emitters so that densely labeled samples can be imaged by acquiring a limited number of frames.

## 3. Approaches to Address Technical Challenges in SRM of Thick Samples with Tissue Clearing

As already underlined, both of these promising sets of techniques are still under rapid development and possess a few flaws with uneven, insufficiently robust labeling, fast fluorescence signal decay and optical aberrations, which is a major issue. In this section we provide a description of how the particular drawbacks of SRM techniques have already been addressed, at least to some extent, by the TOC community. We believe this might serve as a useful guide for researchers planning to perform SRM high-volumetric studies.

### 3.1. Spherical Aberration

The image distortion during thick-tissue imaging that limits the imaging depth of SRM comes both from scattering of light passing through the specimen and the spherical aberration caused by refractive index mismatch between the sample, mounting medium, cover glass/tissue holder and immersion liquid of the objective lens. While, by definition, TOC acts to reduce light scattering, this is not obvious in the case of a spherical aberration that occurs due to refractive index mismatch. Thus, in case of SRM, it is crucial to choose a TOC method that simultaneously offers sufficient clearing and the closest RI match possible in the particular imaging setup.

**Figure 3:**
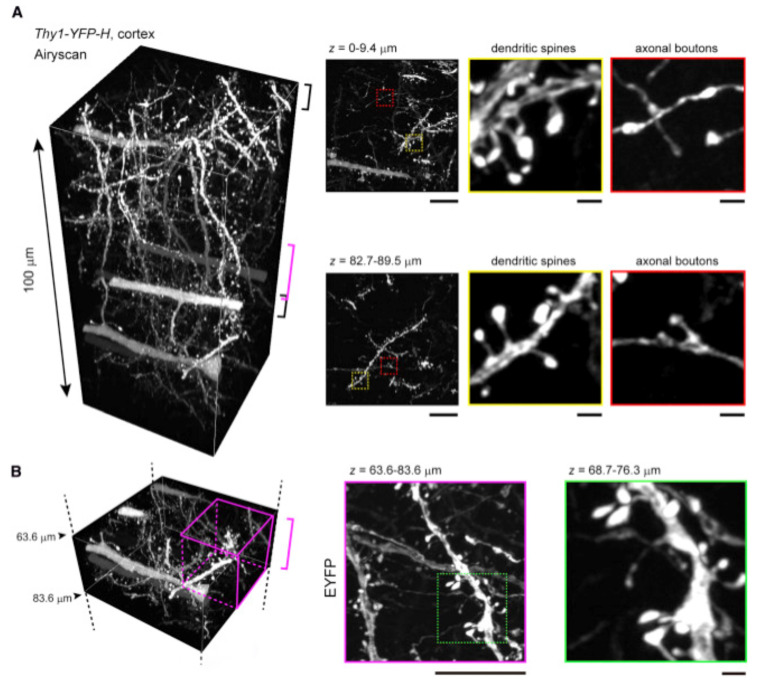
SeeDB2 allows for deep-tissue imaging with SRM resolution. (**A**) Tissue blocks of the cerebral cortex from an adult Thy1-YFP-H mouse were cleared with SeeDB2S; (**B**) upon Airyscan imaging, dendritic spines were identifiable ~100 μm deep into the sample. Reproduced from Ke et al. [58] under the terms of Creative Commons CC-BY-NC-ND license.

The current available data demonstrate that RI ~1.52 and ~1.46 in case of high-NA oil- and glycerol-immersion lenses, respectively, are the most suitable to enhance imaging depth for SRM [55,58] (Table 2). By using novel, iohexol-based clearing and mounting media, Ke et al. [58] achieved SRM imaging conditions (50–150 nm lateral resolution) up to 100 µm deep (Figure 3), with a variety of specimens (HEK293T cells, mouse brain slices and a fly brain) and imaging modalities (STED, SR-SIM, PALM, FV-OSR, SD-OSR, SP8-HyVolution and Airyscan). Importantly, their solutions neither decreased the XFP’s fluorescence nor influenced the size of the samples even after long-term (>1 month) storage. This might be advantageous over the study by Angibaud et al. [55] who, by applying commercial mounting medium CFM3 (RI = 1.518) to 40-µm thick brain slices, achieved a rapid clearing effect (within as little as 5 min of incubation) that substantially improved the depth performance of STED, but at the expense of the expected loss of fluorescence signal intensity.

The adjustment of murine brain tissue RI was also studied by Sawada et al. [43] who examined the applicability of 60% TDE (RI = 1.45) vs. LUCID#2 (RI = 1.496) solutions with a 100× oil-immersion objective for SIM imaging of brain slices. In this comparison, LUCID#2 resulted in superior imaging conditions over TDE, with stable lateral and axial resolutions up to 60- and 40-µm deep, respectively. It should be noted, however, that depending on the residual, postclearing RI of the sample, lower index TOC solutions and objective lenses matched to glycerol might offer better results than the oil immersion. This was recently presented by Bekkouche et al. [59] in the case of deep insect brain imaging, where Rapiclear1.49 and the glycerol-immersion objective was superior to Rapiclear1.52 and the oil-immersion system (with both protocols overcoming results obtained with TDE). Interestingly, they presented additional incubation with the ethanol, before actual TOC and RI-matching, resulting in a tremendous improvement of insect brain transparency.

As the large samples prepared for LSFM must be placed in special imaging cuvettes/chambers, their preparation with materials that match the specific RI of the imaging medium is yet another factor to be taken into account. In a recent work, Glaser et al. [60] presented an extensive list of combinations between TOC methods and compatible materials for the development of cuvettes. Finally, Szczurek et al. [42] point out that the viscosity of the clearing/imaging media should also be perceived as an important factor during SRM, with less viscous solutions obviously penetrating samples better and thus offering advantageous imaging conditions (but at a risk of inducing sample drift when compared to solutions of a higher viscosity [59]).

### 3.2. Suboptimal Intensity of Fluorescence

During imaging, each fluorophore undergoes cyclical shifts between excited and ground states, a number of which depend on the particular fluorophore as well as its chemical environment. With time, the process of photobleaching progressively occurs and causes permanent loss of fluorescence light emission and thus leads to a lowering of both the overall signal-to-noise ratio and image resolution. This is especially true for SIM and STED [8], and several attempts were described to minimize this issue, i.e., triplet state quenching [65], application of low-repetition-rate pulsed lasers [66] and ultrafast scanners [67] or the modification of sample-embedding media [68], the last of which was already exploited in TOC.

Many of the early TOC methods that rendered mammalian tissues highly transparent were based on solvents (e.g., benzyl benzoate, dibenzyl ether [DBE], dichloromethane or tetrahydrofuran [THF]) and suffered from the rapid decay of fluorescence in proteinaceous fluorophores [69,70]. Although it has been widely recognized that rapid, harsh dehydration with THF along with peroxide formation in both THF and RI-matching DBE might contribute to the quenching of XFP’s [61] (green/yellow/red, etc. fluorescent proteins), the modification of these agents was gradual (Table 3). First, Schwarz et al. [71] demonstrated that replacing THF by alkaline tert-butanol (pH = 9.5) during the dehydration step significantly improves fluorescence preservation with optimal imaging conditions being available weeks to months after TOC instead of 24–48 h in the case of other solvent-based approaches available at that time. Although the mechanism of tert-butanol dehydration is far less aggressive than that of other alcohols (including methanol and ethanol), it remains elusive. This might be an additive effect of (1) the kosmotropic nature of tert-butanol (meaning that it stabilizes intramolecular interactions) and (2) the preservation, rather than denaturation, of proteins by tert-butanol [72]. Independent of the mechanism, the application of tert-butanol during the dehydration step inspired Pan et al. [34], who further modified the procedure and included diphenyl ether and tocopherol (a peroxide scavenger) in the final RI-matching solution, naming it uDISCO. While Pan et al. omitted alkalization of the clearing solutions, Li et al. [73] suggested that this might further stabilize the XFP’s signal, an approach that is currently included in the vast majority of modern solvent-based TOC protocols [28,74,75].

**Figure 4:**
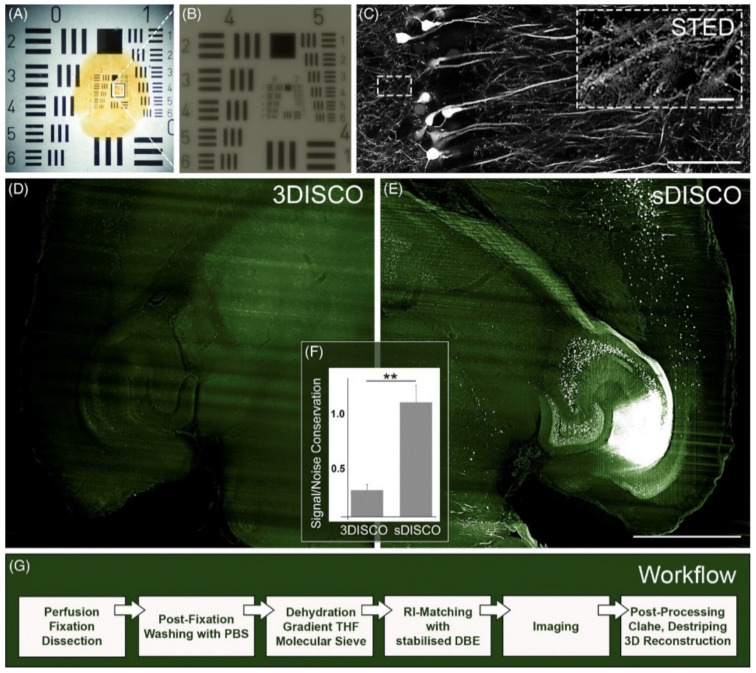
sDISCO greatly stabilizes fluorophores and allows for SRM-based studies. sDISCO achieves transparency of the (**A**) entire mouse brain (**B**) down to 2 μm that is (**C**) compatible with SRM imaging. (**C**) In the overview and detailed image, the thick slice of a Thy1--YFP--H mouse brain was acquired by confocal microscopy using a 40× objective and STED microscopy using a 100× objective, respectively. The scale bars represent 50 μm in the overview image and 5 μm in the inset. (**D**–**F**) sDISCO greatly stabilizes fluorophores even months after the completion of clearing, and (**G**) the effect is achievable with a straightforward protocol. Reproduced from Hahn et al. [61] with permission. ** *p* < 0.01.

It should be underlined, however, that tocopherol is a rather weak scavenger of peroxides, which was recently experimentally verified by Hahn et al. [61]. In a new method, termed stabilized DISCO (sDISCO), this group proved that the removal of aldehydes and peroxides from both dehydrating and RI-matching solutions (i.e., by using column chromatography with basic activated aluminum) greatly enhances the preservation of the fluorescence signal from endogenously encoded fluorophores (Figure 4). Importantly, DBE tends to reaccumulate peroxides and aldehydes, so after their initial removal, 0.4% propyl gallate (an antioxidant broadly used in the food and cosmetics industry) must be added to the final RI-matching solution. This approach allowed Hahn et al. not only to perform successful imaging over one year after the TOC step but also increased the resistance of YFP (in brain tissue derived from a Thy1-YFP-H mouse) to repetitive illumination with STED, which allowed the volumetric visualization of the dendritic spines.

**Figure 5:**
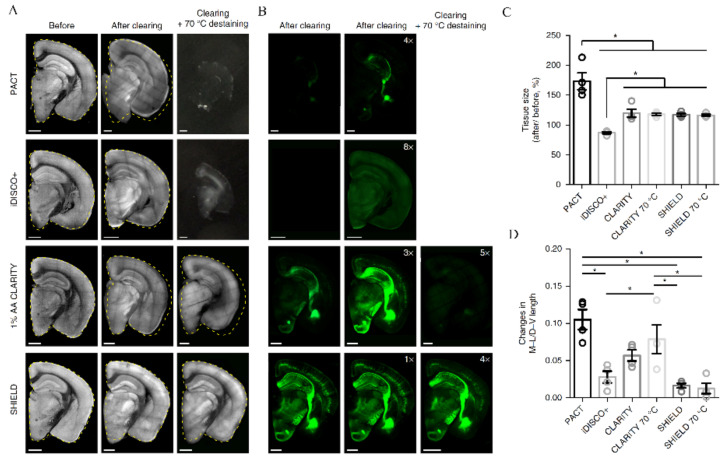
Tissue processing with SHIELD stabilizes its architecture and fluorescence. (**A**) Autofluorescence and (**B**) YFP images of 1-mm-thick mouse brain blocks confirm (**C**,**D**) excellent preservation of fluorescence and tissue size upon SHIELD processing. M-L/D-V length—measurement of mediolateral and dorsoventral lengths of tissue blocks. Reproduced from Park et al. [79] with permission. * *p* < 0.05.

The additional stabilization of fluorophores might be achieved by optimizing the clearing temperature. This was shown several times in the case of CLARITY-related (i.e., tissue transforming) TOC methods [77,78] and once in the case of a solvent-based approach, named FDISCO (DISCO with superior fluorescence-preserving capability), in which THF- and DBE-mediated dehydration and RI-matching, respectively, were performed at 4 °C and led to significantly better preservation of a number of fluorescent proteins and dyes [76]. A completely different approach was recently developed by Chung’s group [79], who pursued identification of a compound that could (i) fully stabilize the fluorescence of proteinaceous fluorophores, (ii) preserve tissue architecture and (iii) maintain both epitopes and transcript integrity for molecular probe labeling. Tissue cross linking during the fixation step with polyglycerol 3--polyglycidyl ether fulfilled the aforementioned criteria and retained XFP’s signal even after a 24 h-long incubation at 70 °C (Figure 5). Such exceptional stabilization along with an easy-to-use protocol that consists of three solutions (SHIELD--perfusion, --OFF and --ON) make it a promising candidate to be applied in SRM samples, where photobleaching serves as an important constraint of the study design.

An alternative approach to the aforementioned methods of preservation of already expressed fluorophores might include the generation of new fluorophore-expressing systems or even the introduction of novel, more stable fluorophores. The former idea was practically presented in brain tissue by Sakaguchi et al. [80], who constructed a new expression system for bright multicolor labeling of neurons. By using a tetracycline response element promoter, instead of a CAG promoter, that was further combined with unmodified XFPs (much brighter than their membrane-bound derivatives), mTurquoise2 (blue), EYFP (green/yellow) and tdTomato (red), in particular, they achieved a sixfold increase in fluorescence intensity. Although the achieved fluorescence was sufficient for high-resolution discrimination of details of neuronal morphology, such as dendritic spines and axonal boutons with a mild TOC technique, SeeDB2 [58], treatment with harsh, solvent-based TOC largely quenched the signal. To overcome this constraint, they introduced the idea of using the genetically encoded chemical tags SNAP, Halo and CLIP and of staining with their respective synthetic labels SNAP-Surface 488, HaloTag TMR Ligand and CLIP-Surface 647, in particular. Such an approach made the fluorescent signal resistant to quenching even with 3DISCO, one of the harshest TOC methods available. Similar, although not epitope-specific, approaches were presented with gold nanoparticles to, e.g., visualize the interactions between nanoparticles and micrometastases [81]. Although the vast majority of approved nanoparticle-based formulations against cancer contain lipids (liposomes and solid lipid nanoparticles, in particular [82]) that are removed with the bulk of TOC, this might be overcome with the application of nanoparticle-conjugated tags that, when arriving in a tissue, remain crosslinked to it even after delipidation [83]. Finally, it should be underlined that new forms of fluorescent proteins are still under development with a few already proven to be stable after being subjected to TOC protocols [84,85,86].

### 3.3. Insufficient and Heterogeneous Molecular Probe Labelling

The extremely high precision offered by SRM requires robust, homogenous labeling of the sample. This has relevance in all microscopy methods but is critically important in the case of SMLM, which samples the distribution of labels stochastically in the entire specimen. As tested by Betzig’s group [87], and recently reviewed by Vangindertael [8], the number of labels and their density that are required to obtain a particular resolution might be five times greater than the Nyquist rate. Thus, in the case of super-resolution imaging of thick samples, it is even more challenging and important to apply labeling methods that guarantee the dense and homogenous detection of particles of interest. Fortunately, this issue was already recognized and extensively studied in TOC and led to several promising, from the perspective of super-resolution microscopists, advancements that currently even allow for whole-body immunolabeling [88].

Generally speaking, most of the advanced ideas that were presented to enhance thick-tissue/organ labeling can be classified into three main categories: (1) pressure-assisted, (2) electrophoresis-driven and (3) chemically modifying of the label affinity (Table 4). Although numerous staining methods have already been optimized for TOC, including nucleic acid stains (DAPI, propidium iodide, SYTO [3]), viral vectors encoding XFP’s [89]; highly effective, bright multicolor labeling systems (i.e., Tetbow [80]); chemical tags and, finally, nanoparticles, we will focus mainly on immunolabeling because of its wide applicability, availability and ease of use. The first report on a robust immunolabeling approach compatible with TOC was iDISCO. In this protocol, Renier et al. [90] took advantage of methanol pretreatment for severe permeabilization and reduction of autofluorescence, an additional bleaching step with H_2_O_2_, followed by treatment with glycine and heparin (which are supposed to reduce tissue background even further). Since its publication in 2014, iDISCO has been successfully applied in dozens of studies, proving its reliability. However, the incompatibility of particular antibodies (not necessarily tissue antigens) with methanol pretreatment is well recognized and should be screened on thin tissue slices before moving forward to the TOC of larger samples. This was partially addressed in the BALANCE method, in which dehydration with ethanol instead of methanol was presumed to prevent deterioration of the less stable epitopes [91]. Moreover, as iDISCO does not utilize any external force to increase the diffusion rate of antibodies, instead utilizing simple diffusion through highly permeabilized tissue, it was reported as incompatible with immunolabeling of densely packed targets, such as neurons activated during learning in the ArcCreERT2 × ChR2-EYFP mice line. In this line, Pavlova et al. [92] reported either an “edge effect” for EYFP-positive cells (saturated labeling on tissue’s periphery with poor staining deep inside) that could not be overcome by titration of the antibody or the enhanced penetration of nanobodies vs. whole IgG antibodies in thick-slices, with the former being incompatible with methanol pretreatment (which severely compromised fluorescent signal) and thus whole-brain labeling. An additional limitation of iDISCO, in view of SRM studies, is the combination of this protocol with solvent-based clearing that, as mentioned above, inevitably causes the tissue volume to shrink (even by 65%), hence lowering the overall resolution during the imaging.

Thus far, the pressure-assisted enhancement of immunolabeling of cleared samples was achieved either by the prolonged perfusion (similar to cardiac perfusion) of antibodies or an application of a syringe pump-based setup. Yang et al. [93] was the first to propose not only the delivery of a TOC solution (4% acrylamide) through the vasculature of a fixed rodent body but also antibodies and small-molecule dyes. Using this approach, named PARS (perfusion-assisted agent release in situ), with the labeling solution being pumped at the constant speed of 1 mL/minute for 3 consecutive days, successful labeling of the mouse brain vasculature and GFAP+ cells (with antibodies directed against mouse immunoglobulin and GFAP, respectively) was achieved. Moreover, PARS was proven effective for peripheral organ staining, with kidneys stained with antitubulin antibody and lectin-based staining of vasculature within the liver, lungs and pancreas. A similar approach, but bearing two important advancements, was presented by Cai et al. [88] in a whole-body clearing approach named vDISCO. In vDISCO (in which v stands for variable domain of heavy chain antibodies), instead of classical, high-molecular weight immunoglobulins (~150 kDa), nanobodies (~15 kDa) were perfused throughout the rodent body to ensure deep tissue labeling. Moreover, perfusion was performed under increased pressure (160–230 mmHg), when compared with standard cardiac protocol (70–110 mmHg). The combination of these two novelties allowed for the visualization of complete neuronal projections of 6-week-old Thy1-GFPM mice (signal boosted with nanobodies) with the immunostaining step taking 9 days (6 days for perfusion and an additional 2–3 days of simple diffusion). It should be added, however, that such unrestricted labeling required two presumably important (as these were not quantified directly) steps, namely cholesterol extraction via addition of methyl-β-cyclodextrin and loosening of the collagen network with trans-1-acetyl-4-hydroxy-L-proline. A similar step of loosening the extracellular matrix via collagenase digestion was recently proposed in a preprint by Biswas et al. [94] who described a rapid, ~3-day long approach for TOC and immunolabeling of entire murine organs. A similar treatment aimed at the digestion of the extracellular matrix with hyaluronidase is a prominent feature of EMOVI (efficient tissue clearing and multiorgan volumetric imaging), a recent TOC pipeline focused on multiplexed antibody-based immunolabeling of immune cells [95].

Although efficient, both PARS and vDISCO are restricted to the labeling of either whole bodies or samples that at least contain large vessels. Another approach, PRESTO (pressure related efficient and stable transfer of macromolecules into organs) is free from this constraint and was published by Lee et al. [96] in two versions: c-PRESTO and s-PRESTO for centrifugal and syringe-based variants, respectively. They observed that either centrifugation of a sample at 600 × g or pressure applied by a syringe filled with antibodies pumped with an infusion/withdrawal of 10 mL per minute significantly increases the penetration depth and rate of antibodies. Although not impressive from a whole-organ imaging perspective, this method might find its place in SRM, as 3 h of c-PRESTO resulted in a 120-μm-deep penetration of labels (a depth that was achievable after 2 days in samples subjected to simple diffusion). A prominent upgrade in the category of pressure-assisted immunolabeling was recently presented by Fiorelli et al. [97], who developed a simple device in which N2 is pumped until 225 kPa is reached in the system, resulting in a fast, uniform labeling across multiple tested tissues and antibodies. More details regarding the construction of the device can be found both in a preprint and their patent description [98].

The first of the tissue-transforming TOC techniques (CLARITY) was developed by the Deisseroth group, in which an acrylamide-bisacrylamide solution created a protein and nucleic acid entrapping mesh [17]. Transparency was then obtainable after harsh, SDS-mediated delipidation, which was further enhanced by an electrophoretic current applied to the sample. Although, soon after the publication, it was recognized that electrophoretic delipidation is a rather unstable, challenging to implement process that often leads to tissue deterioration, the concept to apply electrophoresis to whole-organ TOC was genuinely applied by others. A study by Li et al. [104] demonstrated that while the rate of passive diffusion of antibodies through a CLARITY-prepared brain is inversely proportional to the molecular size of the label (with full IgG’s being the slowest and nanobodies the fastest), it is generally not drastically slowed by the CLARITY mesh. In fact, IgGs penetrated four times slower through the cleared brain slices than through water, while the nanobodies were slowed by only 10%. As the CLARITY mesh did not cause significant prolongation of the immunolabeling process (at least in case of nanobodies and Fab fragments), they chose not to focus on the composition of mesh but to speed up labeling by applying an external voltage of 25 V in a direct current electric field. Such an approach led to a tremendous 800× improvement of labeling in the case of IgGs while keeping the structure of brain cells intact (as verified with confocal microscopy). In order to speed up the process of clearing and labeling and to make these more homogeneous for whole-organ imaging, Kim et al. [99] developed a method of stochastic electrotransport that relies on the continuous rotation of a sample that is placed in a chamber (filled with clearing/staining solution under 15 °C or 4 °C, respectively, to prevent heat-induced tissue damage) between two parallel electrodes. Notably, the application of a stochastic electrotransport technique homogenized both clearing and labeling, with only 1 day required to obtain complete brain immunolabeling with the antihistone H3 antibody. To further increase the labeling rate while keeping the tissue structure intact, Na et al. [100] combined electric and magnetic fields to focus the former in a way that allows it to pass through high-resistant tissue areas with relatively weak electric power (10 W), as compared to previously described electrophoretic approaches. The application of EFIC (electromagnetic focused immunohistochemistry) resulted in the complete immunostaining of CLARITY-precleared 1- and 3-mm-thick brain sections within 3 and 6 h, respectively (using numerous antibodies). Moreover, the utility of such an approach was extended on old, formalin-fixed and precleared human brain samples as well as noncleared rat brain samples, with the latter being stained uniformly through the depth of 800 μm within only 12 h (6 h for the primary and 6 h for the secondary antibody) without any sign of tissue distortion.

**Figure 6:**
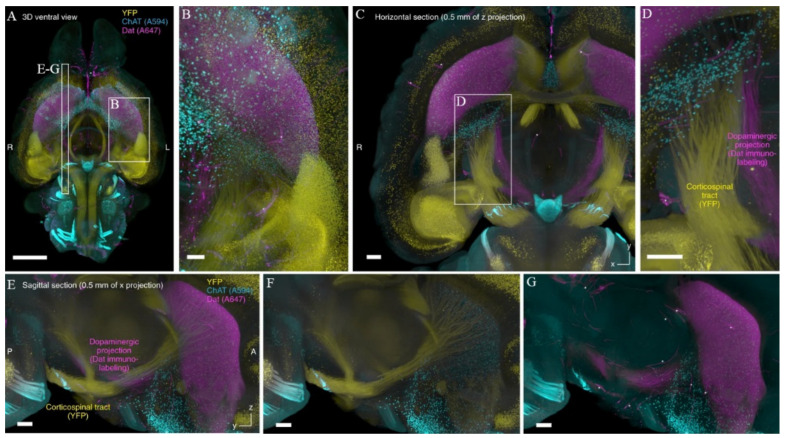
CUBIC-HistoVIsion approach allows for the efficient immunolabeling of the entire mouse brain. (**A**–**D**) The entire murine Thy1-YFP-H brain was cleared, stained using CUBIC-HistoVIsion approach and imaged with the voxel size of 8.3 × 8.3 × 9 μm^3^. The idea that stands behind this pipeline (see the text for details) opens a new way for deep-tissue immunolabeling without the necessity to apply any external forces/apparatus that could potentially lead to sample damage. Images (**E**–**G**) represent reconstituted sagittal sections at the position indicated in (**A**). Reproduced from Susaki et al. [103] under the terms of Creative Commons CC BY license.

Finally, as presented by Chung’s laboratory (inventors of the stochastic electrotransport), homogenous immunolabeling might be further enhanced by modifying the affinity between the probe and its target. In the first of such attempts, Murray et al. [101] proposed two solutions: SWITCH-off (0.5 or 10 mM SDS in PBS) and SWITCH-on (PBST), with an SDS-containing solution vastly preventing antibodies from binding to their respective epitopes. As opposed to control immunostaining of 1-mm-thick mouse brain samples, application of SWITCH solutions resulted in uniform labeling of almost the entire samples of interest stained for 1 day (12 h per solution). A recently presented preprint by Yun et al. [102] is a culmination of formerly published techniques. In a method call eFLASH (electrophoretically-driven fast labeling using affinity sweeping in hydrogel) instead of SDS, a sodium deoxycholate (bile salt)-containing buffer and a change of its pH are used to control the affinity of antibodies. In brief, high concentrations of sodium deoxycholate at a basic pH prevents antibody binding, while low concentrations of sodium deoxycholate at a neutral pH promotes this process. Luckily, when combined with the stochastic electrotransport, both the concentration of bile salts and pH can be changed gradually with ease, starting from low-binding conditions and increasing with time (as the probes penetrate through the sample). Using this approach, Yun et al. overcame the already discussed “edge effect” which, in case of whole-organ immunolabeling, cannot be simply surmounted by saturating the reaction by providing a sufficient amount of antibodies to cover all of the target epitopes. It should be underlined, however, that prior to eFLASH, samples were subjected to another original protocol of this group, called SHIELD. In total, although the entire procedure seems challenging to apply, the commercialization of ready-to-use kits and machinery (that has already taken place in the case of stochastic electrotransport) should bring the great potential that it holds to a wide audience and make the robust, uniform labeling of entire murine organs for organ-wide SRM studies feasible. Recently, Susaki et al. [103] found that PFA-fixed and delipidated tissues (as tested on brain, kidney, liver and muscles) act as an electrolyte gel that expand in alkaline solutions, shrink when put into a highly ionized state and shrink again under an acetone fraction. This observation laid a foundation for the description of completely new protocols that focus on uniform immunolabeling of the entire mouse brain without the necessity to apply any external forces or devices (Figure 6). In brief, the authors present convincing data that immunolabeling within the entire mouse brain can be achieved with special attention paid to three steps: (1) modulation of the interaction between antibody and tissue (with 10% Triton X-100 or Quadrol), (2) avoidance of a two-step staining approach (by application of either a dye-conjugated primary antibody or staining with preformed complexes of primary and dye-conjugated secondary antibody) and (3) taking care of the appropriate staining conditions (i.e., staining in above room temperature, with appropriately high antibody concentration and, applied with special caution, with limited tissue digestion with hyaluronidase or collagenase). Undoubtedly, the application of such a pipeline would require significant, antibody-dependent optimization processes, but after that, it should be perceived as a promising tool to overcome the “edge effect” and thus allow for true whole-organ, quantitative studies to be performed.

## 4. Limitations of TOC in the Context of SRM

Even with such significant progress in the field of TOC, the path towards the imaging of entire rodent organs with SRM, or at least close to SRM, will not be straightforward. One should bear in mind that seemingly unrestricted access to the organ’s volume comes with a few obstacles, all of which are more or less connected to the first and obvious one: the long time required for imaging. This means that the sample must be stable in the TOC solution and, reversibly, the TOC solution must be stable under the imaging conditions. Although the stability of fluorophores with the novel XFP-preserving TOC approaches described above should not be a critical issue to be considered, the possibility that TOC solution influences (and changes over time) the sample’s shape definitely should. Thus far, the vast majority of TOC methods have exerted some influence on tissue size [20,33,105]. While this is not disqualifying, per se, if the effect is known and isotropic, even the slightest but continuous change in tissue morphology during SRM imaging would extremely complicate data analysis or introduce significant bias. Hence, although short TOC protocols are desirable and in demand, complete transparency, as inspected by eye, does not necessarily mean that the clearing process (and change in tissue morphology) is complete [61]. This might be especially true for TOC methods that, by inducing tissue swelling, transform them into softer, less stable samples during handling and mounting for the imaging [106]. Obviously, this is not the case during the organic solvent-based TOC that makes organs more rigid, but also shrunken. This physically decreases the resolution and makes SRM more challenging. Last but not least, many TOC protocols rely on highly saturated sugar solutions (e.g., CUBIC, FOCM [107], FRUIT [108], UbasM [109]) that tend to precipitate either in slightly lower than room temperature conditions, which are usually present in microscopy facilities, or due to water evaporation during prolonged laser-induced heating. Moreover, a change in temperature of the TOC solution during imaging might also affect its RI, an issue to be considered during especially long imaging sessions.

## 5. Selected Applications of TOC in SRM

Although not flawless, TOC has already aided research at nanoscale resolution. By combining 3D-SIM with a TOC reagent specifically developed for this method, referred to as LUCID, Sawada et al. [43] increased the imaging depth in thick mouse brain slices from a few micrometers to over 60 µm and used it to visualize dendritic spines. Imaging with SIM revealed an altered distribution of dendritic spine forms in layer V pyramidal neurons in layers II/III located in the medial prefrontal cortex of mice treated with dexamethasone to induce depression-like behaviors, an effect that was imperceptible by high-NA confocal imaging, as it failed to detect the smallest spines.

TOC with Mowiol 4-88 was indispensable for Sauerbeck et al. [62] to describe SEQUIN (synaptic evaluation and quantification by imaging nanostructure), a technique of imaging and data analysis that consists of immunolabeling, TOC and image scanning microscopy (ISM) of pre- and postsynaptic markers and data processing that allows for the localization of synaptic centroid and for the identification of synaptic loci. With SEQUIN, characteristic patterns of synapse loss were presented in murine models of tauopathy, amyloidosis and diffuse traumatic brain injury, proving the reliability of this new approach to advance studies of the synaptome.

**Figure 7:**
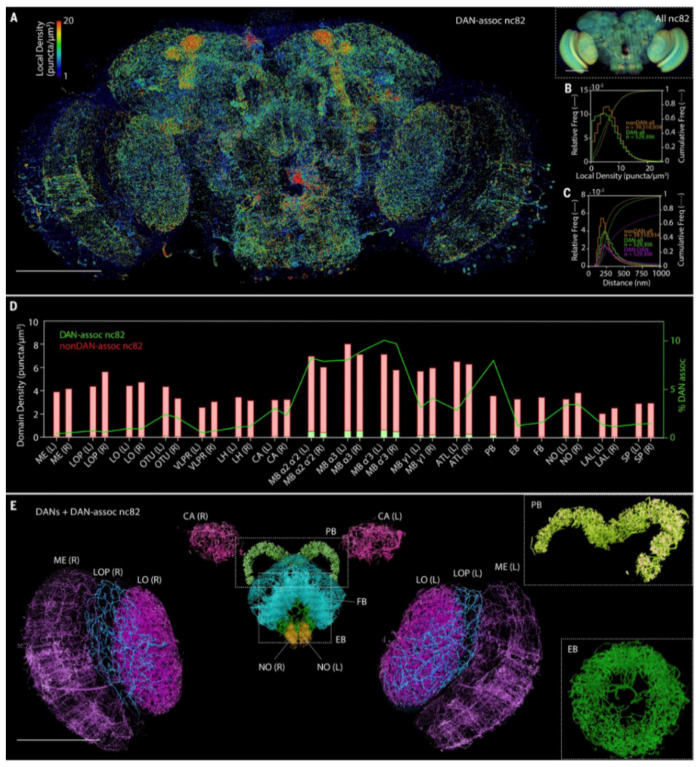
Whole-brain analysis of presynaptic sites and DANs in *Drosophila*. (**A**) MIP view of the subset of nc82 puncta marking presynaptic sites that are associated with DANs (DAN-assoc nc82), color coded by the local puncta density, in an adult *Drosophila* brain. Scale bar, 100 μm. (Inset, right) MIP view of all nc82 puncta, using identical color coding of local density. Scale bar, 100 μm. (**B**) Distribution of local densities of (green) DAN-associated nc82 puncta and (orange) nonDAN-associated nc82 puncta in (**A**). (**C**) Distribution of distances from DAN-associated nc82 puncta (green) and nonDAN-associated nc82 puncta (orange) to the nearest nc82 punctum of any kind, and nearest-neighbor distances from one DAN-associated nc82 to another (magenta). (**D**) Volumetric density of DAN-associated nc82 puncta (green bars) and nonDAN-associated nc82 puncta (red bars), and the percentage of nc82 puncta that are DAN associated (green curve), within each of the 33 brain regions of the adult *Drosophila* brain. (**E**) MIP view of DANs and DAN-associated nc82 puncta, color coded by 13 representative brain regions. Scale bar, 100 μm. (Insets) Magnified views of the (top, angled view) PB and (bottom) EB. Brain regions are ME, medulla; LOP, lobula plate; LO, lobula; OTU, optical tubercle; VLPR, ventrolateral protocerebrum; LH, lateral horn; CA, calyx; MB, mushroom body; ATL, antler; PB, protocerebral bridge; EB, ellipsoid body; FB, fan-shaped body; NO, noduli; LAL, lateral accessory lobe; and SP, superior protocerebrum. “L” and “R” indicate the left and right hemispheres of the brain, respectively. From Gao et al. [11]. Reprinted with permission from AAAS.

Synapses served as a key interest in two other works [11,12], where expansion microscopy and FocusClear, respectively, were used to generate maps of synapse distribution in *D. melanogaster* brains with a submicron resolution. Lin et al. imaged the entire depth of the fly brain with a spinning disk system that guaranteed subdiffraction conditions in the lateral (20 nm) but not axial axis (1 µm). Gao et al. demonstrated that better results in terms of resolution, imaging depth and photobleaching can be achieved with lattice light-sheet microscopy. Using a customized lattice light sheet setup and a relatively low expansion ratio of 4× to avoid tissue morphology distortions, they imaged the whole transgenic fly brain with a sub-100-nm resolution (Figure 7).

Recently, Mizrachi et al. [64] showed that the resolution of LSFM imaging of CUBIC-cleared mouse brain tissue can be combined with SOFI to obtain a 50-nm lateral resolution. Notably, commonly used Alexa fluorophores without specialized buffers were sufficient to observe blinking, which is a prerequisite for SOFI analysis. This allowed for the measurement of axon thickness, the study of dendritic spine morphology and the visualization of single AMPA receptors.

In a recent preprint, Xu et al. [110] aimed to generate a projection map from viral-labeled thalamic neurons of the entire rhesus macaque brain with a close to subdiffraction resolution. Thrillingly, this was achievable within less than 100 h at 1.0 µm × 1.0 µm × 2.5 µm resolution by using serial sectioning (300-µm-thick brain sections) followed by TOC with new solution called PuClear (that utilizes the main chemical used in CUBIC and SeeDB2 and has RI of 1.52).

Finally, a combination of TOC and deep super-resolution imaging is not restricted solely to brain tissue, with Unnersjö-Jess et al. [111,112] reporting on the applicability of expansion microscopy techniques to study podocyte membranes, and scientists from other fields, such as hepatology, already discussing its potential, a valuable application to other peripheral organs [113].

## 6. Summary and Outlook

The application of TOC for SRM studies opens new research opportunities that already allow for the SRM imaging of entire cells and even fly brains, which might be extended to entire rodent or even primate [110] organs in the future. A successful combination of these techniques, however, requires in-depth knowledge regarding the limitations of particular SRM techniques and characteristic features of the TOC to be exploited. It must be underlined that TOC solutions not only can reduce light scattering and spherical aberrations while increasing the signal-to-noise ratio and photostability of fluorophores, but they can also decrease the amount of light-absorbing molecules (the presence of which could severely compromise image quality during deep-tissue SRM imaging). One of the latest discoveries from Dodt’s group addressed this issue in a method called DEEP-Clear (DEpigmEntation-Plus-Clearing), in which several alternative approaches were presented for the efficient removal of natural pigments, such as melanin, ommochromes and pterins [114]. An additional advantage of DEEP-Clear is the ease of application, as it relies on sample soaking in clearing solutions without any external equipment. Moreover, its possible application is further broadened by the compatibility with whole-mount fluorescence RNA in situ hybridization. Other light-absorbing particles, such as hemoglobin or calcified structures (bones or necrotic areas), can also be easily removed by the application of Tetrakis [3] or EDTA [36] during the clearing process, respectively.

Overall, it seems that TOC, in the context of SRM-based studies, offers far more advantages than limitations. However, even perfectly aligned TOC and SRM techniques will be too slow and laborious to perform routine measurements of entire organs with nanometer resolutions, especially if the expansion microscopy approach be used for clearing purposes. Thus, additional major advancements in the field of LSFM, such as tiling LSFM [115] or light-sheet localization microscopy for clarified tissue [116] to speed up the imaging process, are more than awaited along with novel, robust systems for data annotation [117].

## Figures and Tables

**Table 1:** Summary of the selected SRM techniques.

SRM Technique	Acronym	Principle	Major Advantages	Major Disadvantages	Lateral Resolution (nm)	Axial Resolution (nm)
total internal reflection microscopy [46]	TIRF	evanescent wave selectively illuminates and excites fluorophores in a restricted region of the specimen	inherent Z- sectioning	only the outer layer of the specimen can be imaged	200–300	~100
4Pi microscope [47]	4Pi	increase of the effective numerical aperture by using two opposing lenses	high resolution in 3D representations	complex setup	~110	100-150
structured illumination microscopy [48]	SIM	illumination of the sample with a structured pattern generated from a coherent light source	fast, does not require special sample preparation	requires refractive index homogeneity along the optical path	100–130	300-400
super- resolution optical fluctuation imaging [49]	SOFI	correlations between adjacent pixels are calculated to separate signal coming from different fluorophores	no setup modifications, easy to combine with other modalities	requires special fluorophores	100–130	300-400
stimulated emission depletion microscopy [50]	STED	selective deactivation of fluorophores by stimulated emission in donut-shaped region spatially restricts fluorophores spontaneous emission	high lateral resolution	sample is prone to photodamage	~50	~150
single molecule localization microscopy [51,52]	SMLM	controlled switching on/off the fluorophores	high localization precision, relatively easy to upgrade using existing hardware	lower time resolution compared to other SRM techniques, complex sample preparation, challenging in vivo imaging	~20	~50
expansion microscopy [53]	ExM	isotropic swelling of a sample using polymers	compatible with standard imaging and staining techniques	nonuniform expansion of some biological structures, expansion affects fluorophore’s structure	25–70	~200

**Table 2:** Summary of biomedical studies in which TOC and SRM methods were already combined.

TOC	Microscopy	Objective	Specimen	Observable Depth and Achievable Resolution	Time of Clearing
CFM3 (RI 1.518) [55]	STED	100×, NA 1.40, oil	40-μm-thick mouse brain sections	sufficient resolution for detection of dendritic spine necks (which are known to be thinner than the diffraction limit) at 40-μm depth	5 min
SeeDB2S (RI 1.518) [58]	STED	63×, NA 1.40, WD 0.19, oil	thin mouse brain slices	sub-diffraction images up to ~120 μm in depth (limit set by the WD of an objective lens)	a few hours (for relatively thin samples) to 2 days (adult half brain samples)
	SR-SIM	100×, NA 1.46, WD 0.11, oil	HEK293T cells (~10-μm-thick)	HEK293T cells labeled with membrane EGFP, MitoTracker, and DAPI could be fully resolved	a few hours (for relatively thin samples) to 2 days (adult half brain samples)
SeeDB2G (RI 1.46) [58]	confocal	63×, NA 1.30, WD 0.30, glycerol	entire fly brain (~300-μm-thick)	comprehensive maps of *bsh*-positive neurons in a whole brain	a few hours (for relatively thin samples) to 2 days (adult half brain samples)
LUCID#2 (RI 1.496) [43]	SIM	100×, NA 1.49, oil	150-μm-thick mouse brain sections	at a depth of 10 μm, lateral and axial FWHMs were 163 ± 1 and 583 ± 4 nm; maintained at depths from 10 to 60 μm	6 h
Rapiclear 1.49 (RI 1.49) [59]	confocal	63×, NA 1.30, oil	fly brains (freshly dissected dimensions ~3000 by 1500 by 400 μm)	sufficient to capture very fine neurites (diameter of between 136 and 271 nm) up to ~100-μm depth	5 h of ethanol pretreatment + overnight incubation in Rapiclear
H71VE (RI ~1.50) [42]	SIM	63×, 1.40 NA, oil	10-µm-thick paraffin-embedded mouse spleen tissue sections	average modulation contrast-to-noise ratio = 10.1, that remained constant across the entire imaging depth	~1 h
sDISCO (RI 1.56) [61]	STED	100×, NA 1.40, oil	600-μm-thick mouse brain sections	sufficient to visualize single dendritic spines; depth was not studied	days
Mowiol 4-88 (RI 1.46) [62]	image scanning microscopy implemented with the Airyscan microscope	63×, NA 1.40; or 100×, NA 1.46; oil	~50-μm-thick mouse brain sections	140 × 140 × 350 nm (XYZ) at ~50-μm depth	Not described
protein -retention ExM [11]	lattice light-sheet microscopy	25×, NA 1.10, WD 2.00	*D. melanogaster* brain	~60 × 60 × 90 nm for 4× expansion in the entire fly brain	Few days
FocusClear (RI 1.45) [12]	DMI6000 microscope equipped with CSU spinning disk confocal scan head	63×, NA 1.3, glycerol	*D. melanogaster* brain	intact fly brain with 20-nm lateral resolution at ~200-μm depth	1–2 days
Modified CUBIC (mCUBIC described in [63]) [64]	light-sheet fluorescence microscopy and super-resolution optical fluctuation imaging	16×, NA 0.80, WD 3.00	300-μm-thick mouse brain sections	50 × 50 nm lateral pixel size	1 day

**Table 3:** List of solvent-based TOC methods that stabilize fluorophores.

TOC Method Name/Acronym	Key Chemical	Anti-Bleaching Step	pH	Compatible Fluorophores
FluoClearBABB [71]	tert-butanol	not included	9.5	eGFP and mRFP (imaging 271 days after TOC)
uDISCO [34]	tert-butanol, diphenyl ether	tocopherol	not adjusted	GFP (imaging 35 days after TOC), RFP, Texas Red, AF 568 and 647
a-uDISCO [73]	tert-butanol, diphenyl ether	tocopherol	9.0–9.5	GFP (imaging 42 days after TOC)
2Eci [74]	1-propanol, ethyl cinnamate	not included	9.0	GFP, mCherry, AF 488, 568 and 647, Brainbow (GFP, RFP, CFP and YFP)
Eci [75]	ethanol, ethyl cinnamate	not included	9.0	eGFP, eYFP, AF 647
PEGASOS [28]	tert-butanol, Quadrol, poly(ethylene glycol) methacrylate, benzyl benzoate	not included	9.0	eGFP, tdTomato, FITC, AF 488, 568
sDISCO [61]	tetrahydrofuran, dibenzyl ether	propyl gallate, cleared and stored at 4 °C	dehydration in THF in PBS (pH = 8.3)	eGFP, YFP, tdTomato
FDISCO [76]	tetrahydrofuran, dibenzyl ether	cleared and stored at 4 °C	THF and dibenzyl ether of pH = 9.0	GFP, YFP, LEL-Dylight649, Cy5, tdTomato

**Table 4:** TOC methods that increase depth and homogeneity of molecular probe labeling.

Main Mechanism	Acronym	Key Features	Sample	Antibodies/Markers Tested	Time of Procedure
pressure-assisted	PARS [93]	continuous perfusion of clearing agents and antibodies at 1 mL/min rate	mouse body and rat brain	GFAP nanobody (rat brain), anti-mouse IgG antibody (mouse brain), antitubulin (mouse kidney)	3 weeks
pressure-assisted	vDISCO [88]	continuous perfusion of clearing agents and antibodies under increased pressure (160–230 mmHg)	mouse body	updated list of anti/nanobodies available at http://www.discotechnologies.org/vDISCO/	3 weeks
pressure-assisted	PRESTO [96]	centrifugal, c-PRESTO and syringe-based, s-PRESTO, variants available	mouse kidney, lung, liver, testis	anti-collagen type IV, acetylated tubulin, and laminin Abs	1–2 days
pressure-assisted	pIHC [97]	IHC with use of N2 at 225 KPa	up to 1-mm-thick mouse and human brain samples	numerous, e.g., Olig2, Ki67, Iba1, NF, MAP2, NeuN, Lectin, GFAP	hours to 3 days, depending on sample thickness
digestion of extracellular matrix	EMOVI [95]	saponin-based fixation and hyaluronidase-based matrix digestion	various mouse organs (entire, halves and thick slices)	numerous Abs (related to immunology) were tested: anti-CD11c, CD3, CD21, MHCII, LYVE-1, CD31.	6–9 days
digestion of extracellular matrix	SUMIC [94]	collagenase A-based matrix digestion	thick slices of mouse organs and human endocrine gland tissue samples	over 35 Abs tested and verified as compatible	2–3 days
Electro- phoresis	eTANGO [99]	continuous rotation of sample that is placed between two parallel electrodes	entire mouse organs (brain, intestine, heart)	Dylight 594-conjugated tomato lectin	1 day + clearing
Electro- phoresis	EFIC [100]	magnetic force focuses the electric field by bending it onto the sample	CLARITY pre-cleared 1-mm-thick and not cleared, 150-μm-thick, rodent brain samples	numerous Abs, e.g., anti-NeuN, Iba1, GFAP, Neurofilament 200, Myelin Basic protein, Parvalbumin	hours + clearing
modification of probe affinity	SWITCH [101]	SWITCH-OFF that inhibits Abs binding (but allows diffusion) and SWITCH-ON that increases Abs binding	100-μm- to 1-mm-thick mouse and human brain samples	numerous Abs validated, possibility to perform 22 rounds of immunostaining and stripping	1–2 days
modification of probe affinity	eFLASH [102]	eTANGO combined with sodium deoxycholate to control the labeling affinity for various antibodies in a concentration- and pH-dependent manner	mouse and marmoset brains, organoids	numerous Abs, e.g., anti-NeuN, Iba1, GFAP, Parvalbumin, ChAT, c-Fos, NPY	1 day + clearing
modification of probe affinity	CUBIC-Histo VIsion [103]	modulation of interaction between Abs and tissue with Triton X-100 or Quadrol, 1-step staining approach, staining in over RT, digestion with hyaluronidase or collagenase	mouse, marmoset and human brain samples	numerous Abs validated	1–8 weeks + clearing
delipidation	iDISCO [90]	Severe, methanol-based permeabilization and delipidation, some Abs incompatible with methanol pretreatment	adult mouse organs, mouse embryos	numerous, list available at https://idisco.info/validated-antibodies/	~week mouse embryo ~month adult mouse brain
